# Cost-Effectiveness of Preventing Loss to Follow-up in HIV Treatment Programs: A Côte d'Ivoire Appraisal

**DOI:** 10.1371/journal.pmed.1000173

**Published:** 2009-10-27

**Authors:** Elena Losina, Hapsatou Touré, Lauren M. Uhler, Xavier Anglaret, A. David Paltiel, Eric Balestre, Rochelle P. Walensky, Eugène Messou, Milton C. Weinstein, François Dabis, Kenneth A. Freedberg, for the ART-LINC Collaboration of International Epidemiological Databases to Evaluate AIDS (IeDEA), and the CEPAC International investigators

**Affiliations:** 1 Division of General Medicine, Massachusetts General Hospital, Boston, Massachusetts, United States of America; 2 Department of Orthopedic Surgery, Brigham and Women's Hospital, Boston, Massachusetts, United States of America; 3 Department of Biostatistics, Boston University School of Public Health, Boston, Massachusetts, United States of America; 4 INSERM U897, Institut de Santé Publique d'Epidémiologie et de Développement (ISPED), Université Victor Segalen, Bordeaux, France; 5 Centre de Prise en charge, de Recherche et de Formation (CePReF), Abidjan, Côte d'Ivoire; 6 Yale University, New Haven, Connecticut, United States of America; 7 Department of Health Policy and Management, Harvard School of Public Health, Boston, Massachusetts, United States of America; 8 Division of Infectious Disease, Massachusetts General Hospital, Boston, Massachusetts, United States of America; 9 Center for AIDS Research, Harvard Medical School, Boston, Massachusetts, United States of America; 10 Department of Medicine, Harvard Medical School, Boston, Massachusetts, United States of America; 11 Department of Epidemiology, Boston University School of Public Health, Boston, Massachusetts, United States of America; Duke University Medical Center, United States of America

## Abstract

**Background:**

Data from HIV treatment programs in resource-limited settings show extensive rates of loss to follow-up (LTFU) ranging from 5% to 40% within 6 mo of antiretroviral therapy (ART) initiation. Our objective was to project the clinical impact and cost-effectiveness of interventions to prevent LTFU from HIV care in West Africa.

**Methods and Findings:**

We used the Cost-Effectiveness of Preventing AIDS Complications (CEPAC) International model to project the clinical benefits and cost-effectiveness of LTFU-prevention programs from a payer perspective. These programs include components such as eliminating ART co-payments, eliminating charges to patients for opportunistic infection-related drugs, improving personnel training, and providing meals and reimbursing for transportation for participants. The efficacies and costs of these interventions were extensively varied in sensitivity analyses. We used World Health Organization criteria of <3× gross domestic product per capita (3× GDP per capita = US$2,823 for Côte d'Ivoire) as a plausible threshold for “cost-effectiveness.” The main results are based on a reported 18% 1-y LTFU rate. With full retention in care, projected per-person discounted life expectancy starting from age 37 y was 144.7 mo (12.1 y). Survival losses from LTFU within 1 y of ART initiation ranged from 73.9 to 80.7 mo. The intervention costing US$22/person/year (e.g., eliminating ART co-payment) would be cost-effective with an efficacy of at least 12%. An intervention costing US$77/person/year (inclusive of all the components described above) would be cost-effective with an efficacy of at least 41%.

**Conclusions:**

Interventions that prevent LTFU in resource-limited settings would substantially improve survival and would be cost-effective by international criteria with efficacy of at least 12%–41%, depending on the cost of intervention, based on a reported 18% cumulative incidence of LTFU at 1 y after ART initiation. The commitment to start ART and treat HIV in these settings should include interventions to prevent LTFU.

*Please see later in the article for the Editors' Summary*

## Abstract 2

Based on data from West Africa, Elena Losina and colleagues predict that interventions to reduce dropout rates from HIV treatment programs (such as eliminating copayments) will be cost-effective.

## Editors' Summary

**Background:**

Acquired immunodeficiency syndrome (AIDS) has killed more than 25 million people since the first reported case in 1981. Currently, about 33 million people are infected with the human immunodeficiency virus (HIV), which causes AIDS. Two-thirds of people infected with HIV live in sub-Saharan Africa. HIV infects and destroys immune system cells, thereby weakening the immune system and rendering infected individuals susceptible to infection. There is no cure for HIV/AIDS. Combination antiretroviral therapy (ART), a mixture of antiretroviral drugs that suppress the replication of the virus in the body, is used to treat and prevent HIV infection. ART is expensive but major international efforts by governments, international organizations, and funding bodies have increased ART availability. According to World Health Organization (WHO) estimates, at least 9.7 million people in low- and middle-income countries need ART and as of 2007, 3 million of those people had reliable access to the drugs.

**Why Was This Study Done?:**

Although ART is an effective treatment for HIV, a large number of individuals who initiate ART do not receive long-term follow-up care. These patients are generally sicker and have a worse long-term outcome than those who receive follow-up care. Loss to follow up (LTFU) is a significant problem that can undermine the benefits of expanding ART availability. Strategies to improve follow up concentrate on bringing lost patients back into the health care system, but such patients often die before they can be contacted. Prevention of LTFU might be a better strategy to improve HIV care after ART initiation, but there is little information available on which specific interventions might best accomplish this goal.

**What Did the Researchers Do and Find?:**

Given the lack of reported data on the actual costs and effectiveness of LTFU prevention, the researchers used a model to estimate the clinical impact and cost-effectiveness of several possible strategies to prevent LTFU in HIV-infected persons receiving ART in Côte d'Ivoire, West Africa. The researchers used the previously developed Cost-Effectiveness of Preventing AIDS Complications (CEPAC) computer simulation model and combined it with data from a program of ART delivery in Abidjan, Côte d'Ivoire. They then projected the clinical benefits and the cost required to attain a given level of benefit (cost-effectiveness ratio) of different LTFU-prevention strategies from the perspective of the payer (the organization that pays all the medical costs to provide care). Several interventions were considered, including reducing costs to patients (eliminating patient co-payments and paying for transportation) and increasing services to patients at their visits (improving staff training in HIV care, and providing meals at clinic times). LTFU was predicted to cause a 54.3%–58.3% reduction in the estimated life expectancy beyond age 37; patients continuing HIV care were predicted to live a further 144.7 months whie those lost to follow up by 1 year after ART initiation were predicted to live only for a further 73.9–80.7 months. LTFU-prevention strategies in the Côte d'Ivoire were deemed to be cost-effective if they cost less than $2,823 (which is 3× gross domestic product per capita) per year of life saved. The efficacy and cost of the different LTFU-prevention strategies varied in the analyses; stopping ART co-payment alone would be cost-effective at a cost of $22/person/year if it reduced LTFU rates by 12%, while including all the LTFU-prevention strategies described would be cost-effective at $77/person/year if they reduced LTFU-rates by 41%.

**What Do These Findings Mean?:**

The findings suggest that moderately effective strategies for preventing LTFU in resource-limited settings would improve survival, provide good value for money, and should be used to improve HIV treatment programs. Although modeling is valuable to explore the costs and effectiveness of LTFU-prevention strategies it cannot replace the need for more reported data to shed light on problems leading to LTFU and the prevention strategies required to combat it. Also, Côte d'Ivoire might not be representative of all West African countries or resource-limited settings. A similar analysis using data from other ART programs in different countries would be useful to provide better understanding of the impact of LTFU in HIV treatment programs. Finally, the research highlights the cost of second-line ART (a new antiretroviral drug combination for patients in whom first-line treatment fails) as a crucial issue. It is estimated that 5% of all people receiving ART in low- and middle-income countries receive second-line ART and these numbers are expected to increase. Second-line ART had major effects on cost-effectiveness, and a reduction in the cost of this treatment is critical in order to guarantee continued access to HIV treatment.

**Additional Information:**

Please access these Web sites via the online version of this summary at http://dx.doi.org/10.1371/journal.pmed.1000173.

- This study is further discussed in a *PLoS Medicine* Perspective by Gregory Bisson and Jeffrey Stringer
- WHO provides information on disease prevention, treatment, and HIV/AIDS programs and projects
- The UN Millennium Development Goals project site contains information on worldwide efforts to halt the spread of HIV/AIDS
- aidsmap, a nonprofit, nongovernmental organization, provides information on HIV and supporting those living with HIV

## Introduction

Antiretroviral therapy (ART) has been proven to be highly effective at reducing HIV/AIDS-related morbidity and mortality in resource-limited settings [1]–[5]. Despite initial concerns about practicality and operational feasibility, major international efforts have been committed to expanding ART availability in sub-Saharan Africa, where two-thirds of the HIV-infected persons in the world reside [6]. As a result of this scaling-up process, the number of HIV-infected persons treated with ART in sub-Saharan Africa increased from only 100,000 in 2003 to 2.1 million by the end of 2007—a 20-fold expansion over 4 y [7]–[12].

The benefits of the ART roll-out have been limited by a substantial problem with loss to follow-up (LTFU) [13],[14]. A large proportion of HIV-infected patients initiating ART—up to 59% in some settings—are lost to follow-up at some point after ART initiation [7],[11],[15]–[18]. Poor retention in HIV care not only can undermine the impact of scale-up, but can also lead to overstating the performance of HIV programs, because individuals lost to follow-up are generally sicker than those who are retained in care and may therefore experience poorer long-term outcomes than those who remain in care [19],[20]. Moreover, mortality estimates produced by active or passive surveillance programs are dramatically different, often leading to overly optimistic results from programs with passive follow-up [13],[14],[21],[22].

Strategies to improve follow-up generally focus on efforts to bring lost patients back into the health care system (e.g., by outreach teams and collaboration with community organizations). These measures to reverse LTFU have shown that many patients identified as lost have already died [22]–[25]. Preventing LTFU may, therefore, be more effective at improving outcomes. Because such prevention programs may require sustained, long-term investment, their economic feasibility is an important issue. Given the lack of reported data on the actual intervention cost and efficacy of LTFU prevention, we frame this analysis as a “what if” study to provide targets, in terms of both efficacy and cost, to understand how the results of interventions to prevent LTFU might fit into the broader context of HIV treatment [26]. We sought to estimate the long-term clinical benefits and cost-effectiveness of several plausible LTFU-preventive strategies among HIV-infected persons receiving ART in Côte d'Ivoire, West Africa.

## Methods

### Analytic Overview

We coupled data from the Aconda program, an Abidjan-based nongovernmental organization providing ART delivery in Côte d'Ivoire [11], with the Cost-Effectiveness of Preventing AIDS Complications (CEPAC) International simulation model of HIV disease and treatment [27]–[29]. We examined the effectiveness and cost-effectiveness of alternative strategies to prevent LTFU in HIV-infected persons initiating ART in Côte d'Ivoire. We considered several interventions with different levels of effectiveness and costs, ranging from solely eliminating patient co-payments for ART, to multimodal interventions that remove co-payment for ART and medications used to treat AIDS-defining illnesses, improve health care provider training, provide meals at clinic visits, and offer transportation reimbursement. We compared these interventions to the current standard of care in Abidjan. We evaluated cost-effectiveness from the payer perspective, wherein all direct medical costs accruing to the organization providing care (e.g., a health center making treatment available) were considered in the analysis.

We used the model to project life expectancy and total costs of care for HIV-infected persons treated with ART and who remained in care, as well as for those who discontinued care at 1, 3, 6, or 12 mo after ART initiation. We estimated the effectiveness of LTFU prevention interventions as the additional survival benefits resulting from fewer HIV-infected persons discontinuing ART prematurely. We estimated the cost-effectiveness of the LTFU prevention interventions as the difference in costs divided by the difference in life expectancy of the strategies, with and without each LTFU prevention intervention. All costs were reported in 2006 US$ using country-specific gross domestic product (GDP) deflators [30] and the 2006 mean exchange rate between the CFA franc and the US$ (US$1 = CFA 540) (GDP and 3× GDP are per capita) [31]. All costs and life expectancies were discounted 3% per year [32]. We utilized guidelines from the Commission on Macroeconomics and Health of the World Health Organization (WHO) to determine whether specific LTFU prevention interventions might be considered “cost-effective.” By these guidelines, an intervention is considered “cost-effective” if its cost-effectiveness ratio is below 3× the per capita GDP in a country (US$2,823 in 2006 for Côte d'Ivoire) and “very cost-effective” if the cost-effectiveness ratio is below 1× the annual per capita GDP (US$941) [30],[33].

### The CEPAC International Model

The CEPAC International model is a widely published computer simulation model of HIV disease that portrays natural history and treatment strategies for HIV-infected individuals in resource-limited settings [27]–[29]. In the model, each patient from a simulated cohort of HIV-infected individuals transitions among health states defined by CD4 count, HIV RNA level, history of opportunistic infections (OIs), and ART use. In the absence of ART, the natural history of HIV disease is determined by CD4 count decline, stratified by HIV RNA level, and CD4-specific rates of HIV morbidity and mortality [34]. Monthly probabilities of OIs and mortality rates are derived from the Agence Nationale de Recherches sur le Sida et les Hépatites Virales (ANRS) 1203 Cotrame and the ANRS 1220 Primo-CI cohorts in Côte d'Ivoire [11],[34],[35]. ART reduces HIV RNA levels, increases CD4 counts, and thereby confers a decrease in OI incidence and HIV-related mortality [36]. In this analysis, the model assumes availability of two lines of ART for persons in care. Patients receive first-line non-nucleoside reverse transcriptase inhibitor (NNRTI)-based ART consisting of nevirapine/lamivudine/stavudine and second-line protease inhibitor (PI)-based ART consisting of lopinavir/ritonovir/tenofovir/emtricitabine [11]. The standard of care includes clinic visits every 3 mo, as well as in the month of any acute OI.

Each simulated patient is assigned a CD4 count and HIV RNA level at the time of entry into HIV care and is followed until the time of death. Results from large numbers of these individual simulations are aggregated to develop stable population estimates of outcomes. Additional details on the model structure have been published elsewhere [37],[38].

### Input Parameters for the Model

#### Cohort characteristics

Initial cohort characteristics in the model are based on the demographic characteristics of HIV-infected patients from the Aconda program sites in Abidjan, Côte d'Ivoire, a cohort participating in the international ANRS/National Institutes of Health (NIH)-funded collaboration ART-LINC of IeDEA [39]. The Aconda program relies on two different types of HIV care delivery: (1) the CePReF clinic, a clinical research study center entirely dedicated to HIV care, and (2) public and private health care facilities, which include general medical care as well as HIV-specific care. In this analysis, we focused on people receiving HIV care at the public and private general health care facilities. Patients receiving HIV treatment at these facilities had a mean age of 37 y (standard deviation [SD], 9 y) and a mean CD4 count of 140/µl (SD, 116/µl) at the time of ART initiation; 70% of them were female (Table 1).

**Table 1:** Model input parameters for an analysis of LTFU from HIV programs in Abidjan, Côte d'Ivoire.

Variable	Value (SD)	References
**Baseline cohort characteristics**		
**Age (y)**	37 (9)	[11]
**CD4 count (cells/µl)**	140 (116)	[11]
**Gender distribution (% female)**	70	[11]
**Lost to follow-up** a **at 1 y (%)**	18	[11]
**Given lost, time at which LTFU occurred (%)**	—	[11]
**0–1 month**	12	[11]
**1–3 mo**	19	[11]
**3–6 mo**	25	[11]
**6–12 mo**	44	[11]
**Efficacy of antiretroviral therapy**		
**Virologic suppression at 24 wk (%)**		
**First-line therapy** b	80.4	Unpublished data
**Second-line therapy** b	80.4	Assumption
**Probability of toxicity occurrence**		
**Minor toxicity**	0.77	[41]
**Major toxicity**	0.05	[41]
**Probability of late virologic failure, monthly** c	0.012	—
**Costs (2006 US$)**		
**First-line ART, monthly** d	4.98	[48]
**Second-line ART, monthly** d	55.98	[48]
**Minor ART-related toxicity, per event**	2.24	[38]
**Major ART-related toxicity, per event**	22.39	[38]
**Co-trimoxazole, monthly**	1.81	[38]
**Routine care (range by CD4 count), monthly**	20.87–28.57	[36],[38]
**CD4 count, per test**	25.00	[37]
**Intervention efficacies examined (% reduction in LTFU)**	10–75	—
**Interventions examined**	—	[47]
**1. Elimination of the ART co-payment (cost/person/year [US$])**	22	[47]
**2. Intervention 1 + providing OI-related medications free to the patient (cost/person/year [US$])**	41	[47]
**3. Intervention 2 + increased training for health care workers (cost/person/year [US$])**	53	[47]
**4. Intervention 3 + reimbursing transportation costs and providing breakfast (cost/person/year [US$])**	77	[47]

a LTFU, patients whose last contact with the care center was at least 3 mo prior and who were not known to be dead or transferred to another care center. Those who were lost to follow-up are assumed to re-enter care upon the occurrence of any Stage 3–4 OI.

b First-line ART, non-nucleoside reverse transcriptase inhibitor-based regimen; second-line ART, PI-based regimen. Efficacies are those reported for the “on treatment” group.

c Patients who have achieved virologic suppression at 24 wk have a continuing risk of later virologic failure.

d Cost does not include monthly co-payment of approximately US$2.

#### Efficacy of ART

Virologic suppression with first-line, NNRTI-based ART is estimated at 80.4% (unpublished data, Côte d'Ivoire). After 12 mo, patients switch to second-line therapy if they experience a severe OI (excluding tuberculosis and bacterial diseases), or if their CD4 count is observed to decrease ≥50% from initial CD4 count or peak on-treatment CD4 [40]. In the absence of specific data from Côte d'Ivoire, we assumed that virologic suppression on second-line, protease inhibitor-based therapy was similar to that of first-line therapy (80.4% at 24 wk). Patients on ART face the possibility of developing minor or major toxicities, and patients who achieve virologic suppression have the possibility of experiencing later virologic failure [41]. We assumed that ART-related toxicities led to a drug substitution but not discontinuation of treatment. In the base case, we assumed that patients continued receiving second-line ART even after clinical failure, until death.

#### Rates of LTFU

Patients were considered lost to follow-up if (1) they were not known to be deceased, (2) they were not known to have transferred to another care center, and (3) for patients receiving ART, the time since last contact with the Aconda site exceeded 3 mo [11]. For the base case estimate of LTFU rates, we used the average cumulative incidence of 18% LTFU after 1 y from the 18 Aconda program general care centers (excluding CePReF) [11]. Among patients lost to follow-up, 12% were lost during the first month, 19% between months 1–3, 25% between months 3–6, and 44% between months 6–12 (Table 1). We used the LTFU rate at CePReF (cumulative 1 y incidence of 11%), as the basis for estimating the LTFU prevention program efficacy, since this is an example of an HIV specialty center with community outreach to patients at risk of being lost to follow-up [21]. We used this reduction in LTFU rates (from 18% to 11%, or 40% efficacy, [18%–11%]/18%) as the anchor for basing the efficacy of the different interventions. We examined a range of 10% to 75% efficacy to assess the impact on the outcomes.

We assumed that patients who were lost to follow-up discontinued ART and experienced an accelerated CD4 count decline until their HIV RNA returned to its pretreatment set point, at which time their CD4 count continued to decline on the basis of the natural history of HIV disease [42],[43]. During this time they were at increased risk of morbidity and mortality compared to those patients not lost to follow-up.

We assumed that HIV-infected patients who are lost re-enter care when they develop a WHO Stage 3–4 OI (excluding tuberculosis and bacterial diseases) [40]. Since those who are lost to care are subject to higher morbidity and mortality, only a fraction of them will be able to return to care. By recognizing that some patients will eventually re-enter care, even in the absence of specific interventions, we deliberately introduce a conservative bias against the LTFU-prevention programs in terms of the benefits derived from retention in care [44].

### Interventions for Preventing LTFU under Consideration

We considered four alternative interventions to prevent LTFU (Table 1). These interventions are incremental in their content: each subsequent intervention adds components and costs to the previous one. The first intervention consists of shifting the costs that patients pay (the patient's co-pay in Côte d'Ivoire of about US$2/month, US$22/year) to the center providing ART. The second intervention also eliminates the cost to patients for medications to treat OIs. Non-ART drugs are currently fully charged to the patients. This cost can impose a major financial burden on HIV-infected patients; a recent study carried out in the same setting revealed that on average the cost of the OI-related drugs represents 11.8% of the family income [45]. These drugs include but are not limited to cotrimoxazole, nystatin, paracetamol, imodium, ibuprofen, and fluconazole [46]. The third intervention adds the resources required to improve HIV care skills among health care workers in public general health centers to the level of those at the HIV-treatment center (CePReF). The fourth intervention adds provision of breakfast or lunch and reimbursement of transportation costs for patients attending scheduled appointments.

The costs of the LTFU prevention programs were derived from the UNAIDS/UNFPA Rapport National de la Côte d'Ivoire as well as personnel costs from the clinics and estimated real costs in Abidjan. The costs per person per year for the four interventions were US$22, US$41, US$53, and US$77 (Table 1 and Text S1 for details) [47].

#### Cost of HIV care

The costs of ART regimens were based on the lowest generic drug prices available for lower-income countries for the most common first- and second-line regimens used in Côte d'Ivoire (US$4.98/month for first-line ART and US$55.98/month for second-line ART, Table 1) [48]. The cost of co-trimoxazole prophylaxis was estimated at US$1.81/month [38]. The cost of a CD4 count was estimated at US$25/test, with tests performed every 6 mo, at scheduled clinic visits [11]. Routine care costs in addition to these specific items include outpatient visits, laboratory tests, procedures including x-rays, and pharmacy [38]. Costs of office visits and hospital stays were obtained from Yopougon University Hospital in Abidjan [38]. The average cost per year per person on ART is US$820, which includes the cost of ART, prophylaxis, clinic visits, and laboratory monitoring, including CD4 cell count.

### Sensitivity Analyses

We varied several key parameters in the model across wide ranges to examine which had the greatest effect on survival, cost, and cost-effectiveness. We examined increases and decreases in the efficacy of both first- and second-line ART, and evaluated different strategies following second-line ART failure, including remaining on failed second-line therapy, discontinuing second-line ART after failure, or recycling first-line drugs. We also examined the assumption that patients who were lost to follow-up re-enter care at the time of a severe OI by considering that those lost to follow-up never re-enter care, that they re-enter care upon the occurrence of any OI, or that they re-enter with CD4 count <50/µl. In addition, we conducted a sensitivity analysis in which we evaluated the role of HIV RNA monitoring on the cost-effectiveness of LTFU prevention interventions. With respect to costs, we increased as well as decreased the cost of first- and second-line ART, the cost of treating OIs, and routine care costs. We also varied baseline rates of LTFU.

## Results

### Per-Person Life Expectancy Losses Due to LTFU

The projected undiscounted life expectancy for an HIV-infected patient initiating ART at age 37 y with CD4 140/µl and remaining in care without LTFU is 201.5 mo (16.8 y, Table 2). For patients lost to follow-up after 1, 3, 6, or 12 mo on ART, projected life expectancy is estimated at 84.0, 87.3, 88.4, or 92.1 mo, respectively. The life expectancy loss due to LTFU therefore ranges from 117.5 mo to 109.4 mo, or 58.3% to 54.3% of life expectancy, depending on the timing of being lost. The projected life expectancy of a typical patient lost to follow-up in the Aconda program is estimated at 89.3 mo (7.4 y), which represents a loss of 112.2 mo (9.4 y) compared to an ideal retention rate of 100%.

**Table 2:** Life expectancy loss due to LTFU in a cohort of patients in Abidjan, Côte d'Ivoire.

Time from ART Initiation until LTFU	Discounted^a^		Undiscounted	
	Life Expectancy (mo)	Life Expectancy lost (mo)	Life Expectancy (mo)	Life Expectancy Lost (mo)
**No LTFU**	144.7	0	201.5	0
**Months following ART initiation**				
**0–1**	64.0	80.7	84.0	117.5
**2–3**	66.5	78.2	87.3	114.2
**4–6**	67.6	77.1	88.4	113.1
**7–12**	70.9	73.9	92.1	109.4
**At Aconda** b	68.4	76.3	89.3	112.2

a Discounted at 3% per year.

b Taking into account timing of LTFU, given that patients were differentially lost to follow-up (12% lost after 1 mo, 19% lost after 3 mo, etc. See Table 1 for details).

### Community Impact of LTFU on Life Expectancy

With an 18% LTFU rate for the 6,704 patients receiving ART through Aconda sites, 1,206 patients are lost by 1 y after ART initiation. If each patient loses an average of 9.4 y of life, the total life expectancy loss for the cohort of HIV-infected patients currently in Aconda community clinics due to LTFU would reach 11,336 y.

### Cost-Effectiveness of Interventions Focused on LTFU Prevention

As the efficacy in preventing LTFU increases, projected discounted life expectancy increases, from 132.4 mo with 10% efficacy to 141.3 mo with 75% efficacy. Because patients remain in treatment longer on average with better retention, total lifetime costs also increase. For the LTFU prevention program costing US$22/person/year, per person lifetime costs range from US$9,100 if the intervention is 10% effective to US$9,900 if the intervention is 75% effective (Table 3). The cost-effectiveness ratios of the LTFU intervention, compared to no intervention, vary depending on the efficacy of the LTFU intervention. For the US$22/person/year intervention the cost-effectiveness ratios range from US$3,100 per year of life saved (YLS) at 10% efficacy to US$1,200/YLS at 75% efficacy. Cost-effectiveness ratios for the other LTFU prevention programs are presented in Table 3.

**Table 3:** Lifetime costs, life expectancy, and cost-effectiveness of interventions to prevent LTFU in Aconda centers, Côte d'Ivoire.

Effectiveness of Intervention (% Reduction in LTFU)	Discounted per Person Lifetime Costs	Discounted per Person Life Expectancy (Mo)	Cost-Effectiveness Ratio (US$/YLS)
**No intervention**	US$8,800	131.0	—
**US$22 intervention (/person/year)**			
**10**	US$9,100	132.4	US$3,100
**25**	US$9,300	134.4	US$1,800
**50**	US$9,600	137.9	US$1,400
**75**	US$9,900	141.3	US$1,200
**US$41 intervention (/person/year)**			
**10**	US$9,300	132.4	US$4,900
**25**	US$9,500	134.4	US$2,600
**50**	US$9,800	137.9	US$1,800
**75**	US$10,100	141.3	US$1,500
**US$53 intervention (/person/year)**			
**10**	US$9,500	132.4	US$6,100
**25**	US$9,600	134.4	US$3,000
**50**	US$9,900	137.9	US$2,000
**75**	US$10,200	141.3	US$1,700
**US$77 intervention (/person/year)**			
**10**	US$9,700	132.4	US$8,400
**25**	US$9,900	134.4	US$4,000
**50**	US$10,200	137.9	US$2,500
**75**	US$10,500	141.3	US$2,000

Baseline LTFU rate is 18%. All cost-effectiveness ratios are computed on an incremental basis using the “no intervention” strategy as the comparator. See Methods and Table 1 for details about each intervention.

### Guidance for the Prospective Evaluation of LTFU Prevention Programs

**Figure 1:**
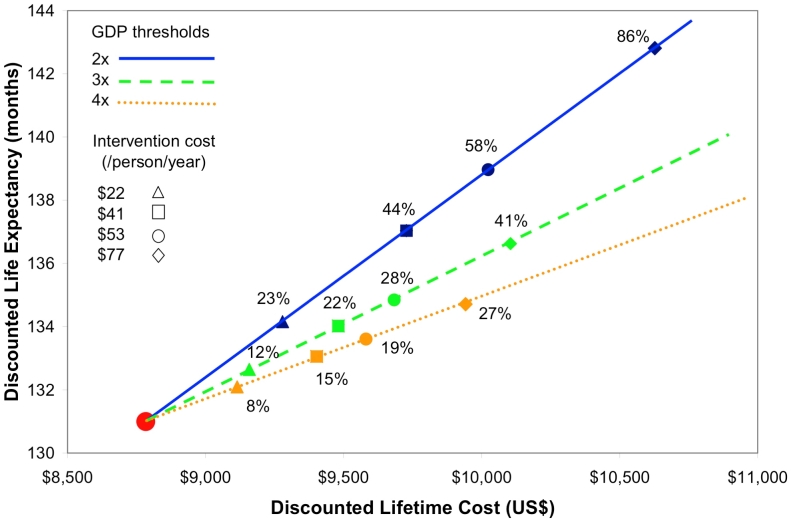
Threshold efficacy, cost, and life expectancy associated with LTFU prevention interventions in Côte d'Ivoire. This figure describes threshold efficacy for alternative willingness to pay thresholds, shown in blue (2× per capita GDP), green (3× per capita GDP), and orange (4× per capita GDP). Triangles represent efficacy thresholds for LTFU interventions at US$22/person/year, squares at US$41/person/year, circles at US$53/person/year, and diamonds at US$77/person/year. The vertical axis shows the per person discounted life expectancy and the horizontal axis shows the per person discounted lifetime cost. The red dot in the lower left corner represents the per person life expectancy and lifetime cost in a program with no LTFU intervention.

Figure 1 shows the minimal efficacy at which a LTFU prevention program might be considered cost-effective using three different cost-effectiveness thresholds: 2×, 3×, and 4× per capita GDP [33]. In settings with 18% LTFU at baseline, and using 3× per capita GDP as a threshold for “cost-effectiveness,” the LTFU prevention program that costs US$22/person/year would be considered cost-effective with an efficacy of at least 12%. LTFU programs that cost US$53 or US$77 would be considered cost-effective at a 3× per capita GDP threshold if they could reduce LTFU rates by at least 28% and 41% (dashed line). At a 4× per capita GDP cost-effectiveness threshold, the LTFU prevention intervention costing US$22/person/year would be considered cost-effective if it could reduce LTFU rates by at least 8%, and the intervention costing US$77/person/year would be considered cost-effective at 27% efficacy (dotted line). At a 2× GDP per capita threshold, these interventions would be considered cost-effective at efficacies of 23% and 86% (solid line).

Furthermore, Figure 1 shows that the intervention that costs US$22/person/year and that provides a 12% reduction in rates of LTFU would provide similar value as the more expensive (US$77/person/year) intervention that is 41% efficacious, but the more expensive strategy would provide more benefits compared to the less expensive intervention, increasing overall life expectancy by 5.6 versus 1.6 discounted mo (unpublished data).

### Sensitivity Analyses

**Figure 2:**
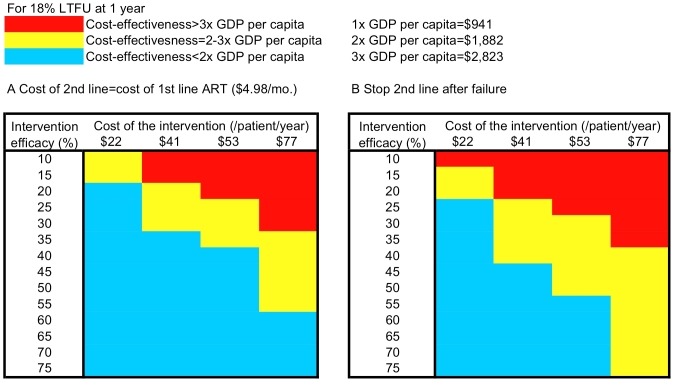
Sensitivity analysis on cost and efficacy of interventions to prevent LTFU with 18% baseline LTFU. (A and B) Represent the cost-effectiveness of LTFU prevention strategies as a function of cost (columns) and efficacy (rows). (A) Illustrates the scenario where the cost of second-line ART is decreased to the cost of first-line ART (US$4.98/month, excluding patient co-payment). (B) Shows the scenario of stopping second-line ART after failure instead of continuing ineffective therapy. The light blue areas represent combinations of cost and efficacy of LTFU prevention strategies under each ART cost composition that ensure cost-effectiveness of LTFU strategies below 2× per capita GDP. The yellow area represents combinations of cost and efficacy of LTFU interventions that produce cost-effectiveness ratios between 2× and 3× per capita GDP. The red area represents scenarios where the cost-effectiveness ratios of LTFU interventions exceed 3× per capita GDP.

We varied key parameters in the model to determine those with the biggest impact on the results. We found that the cost of second-line ART is the most important factor affecting the cost-effectiveness of LTFU prevention interventions. Reducing the cost of second-line ART to that of the cost of first-line ART (US$4.98/month) decreased all cost-effectiveness ratios, so that the US$41/person/year intervention would be cost-effective by the <3× per capita GDP criterion, if it were at least 20% effective (Figure 2A). When we assumed that patients stopped treatment after second-line ART failure, instead of assuming that they continued for the rest of their lives, the US$41/person/year intervention would be cost-effective by the <3× per capita GDP criterion, provided that it was at least 25% effective (Figure 2B).

Sensitivity analysis with HIV RNA monitoring available resulted in slight increases in the cost-effectiveness ratios of LTFU prevention strategies. The cost-effectiveness ratios increased by 6%–20%; the magnitude of the increase depended on the efficacy and cost of the intervention, but the ratios remained within the same thresholds for cost-effectiveness as the base case analysis results.

**Figure 3:**
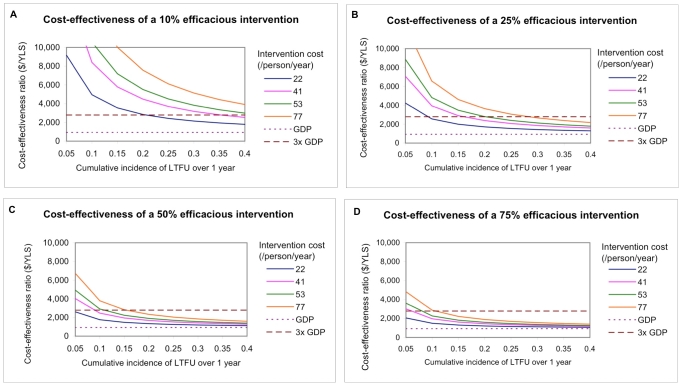
Cost-effectiveness of interventions to prevent LTFU, stratified by intervention cost. This figure shows the cost-effectiveness ratios of interventions ranging in efficacy from 10%–75%, stratified by cost (US$22, US$41, US$53, and US$77/person/year) and at cumulative incidences of LTFU ranging from 5%–40% over 1 y.

The baseline rate of LTFU affects the cost-effectiveness of LTFU prevention interventions. Figure 3 shows the cost-effectiveness of four interventions of various efficacies and costs at cumulative incidences of LTFU ranging from 5%–40% over 1 y. For example, in a program with 20% LTFU over 1 y, an intervention with 10% efficacy at preventing LTFU that costs US$22/person/year has a cost-effectiveness ratio less than 3× per capita GDP (Figure 3A). In the same setting, if the efficacy of the LTFU intervention was increased to 25%, an intervention costing US$41 or US$53/person/year would have a cost-effectiveness ratio less than 3× per capita GDP (Figure 3B). At 50% or 75% efficacy of reducing LTFU, an intervention of any cost considered (US$22–US$77/person/year) would have a cost-effectiveness ratio less than 3× per capita GDP (Figure 3C, 3D).

We also varied the assumptions about the behavior and outcomes of patients who are lost to follow-up. In the base case, all patients who are lost to follow-up re-enter care upon the occurrence of a severe OI. This assumption results in 56.4% of those lost to follow-up dying without returning to care. If all patients re-enter care with any OI, the cost-effectiveness ratio for the US$41/person/year intervention with 50% efficacy increases from US$1,800/YLS to US$4,200/YLS. If all patients lost to follow-up re-enter care when their CD4 count drops below 50 cells/µl, the cost-effectiveness ratio of the US$41/person/year intervention (at 50% efficacy) is equivalent to that of the base case (US$1,800/YLS). If those who are lost to follow-up never re-enter care, their life expectancy is decreased to 2.5 y and the cost-effectiveness ratio decreases to US$1,400/YLS.

## Discussion

Using a simulation model for HIV treatment in Abidjan, Côte d'Ivoire, we estimate that LTFU from HIV treatment programs leads to substantial losses in projected life expectancy—112.2 mo on average for those lost during the first year of treatment. For a program with about 6,700 patients, like the Aconda program in Abidjan, these per patient numbers translate into over 11,000 y of life lost—more than 1.5 y of life lost per person in the program. A substantial proportion of these lost years of life could be saved by implementing an effective LTFU prevention intervention. While evidence on the efficacy of interventions to prevent LTFU is only now beginning to accumulate [49], we find that such interventions, if they cost less than US$22–US$53/person/year, would be highly cost-effective under plausible assumptions of efficacy.

ART treatment programs in many resource-limited settings are now reporting increasing problems with LTFU [14],[19],[50]. Evidence is also accumulating with regard to the causes of LTFU in resource-limited settings and programs to reduce LTFU have focused on either reducing costs to patients (ART co-payments, OI costs, transportation costs) or increasing the benefits to patients for their visits (free lunch, improved service because of better trained personnel). Although the WHO has recommended free access to ART, many countries have been unable to deliver such programs [51],[52]. Data from the ART-LINC Collaboration, including the cohorts in Côte d'Ivoire, as well as programs in Botswana and Tanzania, have found that charging a fee for care is associated with increased LTFU and increased mortality [13],[50],[53],[54]. Recent data from Cameroon show that patients' self-reported financial difficulty in purchasing ART was associated with lower adherence and lower CD4 count after 6 mo on treatment [55]. In a meta-analysis on LTFU from ART programs, Rosen et al. found that programs that did not require patients to pay for treatment had higher retention rates at 6 mo compared to programs requiring partial or full payment [14]. Recent data from Kenya suggest that providing ART free to patients led to a 57% decrease in LTFU [56]. On the basis of these recent data from Kenya, in August 2008, the government of Côte d'Ivoire mandated ART free to all patients [57]. Transportation costs may also play an important role in determining outcomes in those living farther from their site of care [58]–[60]. Studies in Burkina Faso and Mali found decreased viral suppression in patients living farther from the clinic, suggesting follow-up as a major determinant of success [61]. Food insecurity also poses a challenge to HIV-infected patients who have competing priorities of securing food as well as HIV treatment. Providing food at the time of the clinic visit may encourage some patients to attend their clinic appointment or to get their medications [62].

For example, the CePReF center in the Aconda program, which utilized a team of social workers and people living with HIV/AIDS to make telephone calls or home visits to patients who did not keep their scheduled appointments, had rates of LTFU at 1 y that were 40% lower than the general health care centers in the program. At an intervention cost of US$22–US$53/person/year, the cost-effectiveness ratio of such an intervention is less than US$2,800/YLS, which is <3× the per capita GDP in Côte d'Ivoire.

Since the efficacy of each individual component of the LTFU prevention interventions considered in the analyses reported here is unknown, we have ordered the interventions in terms of what may be most effective and feasible. However, parts of each intervention (or interventions not specified here) could be implemented individually or in different combinations than we proposed. We prioritized eliminating ART co-payments as the first step because this has been previously shown to be associated with better outcomes in several studies in Africa, and because it is increasingly considered feasible.

The LTFU prevention interventions considered in this analysis focused on reducing costs to the patient and improving health care workers' skills, but other strategies to prevent LTFU may also be considered as part of an effective LTFU prevention intervention. For example, health care workers may send patients text messages to remind them of their upcoming clinic appointments, or make phone calls to patients who have recently missed appointments. Another possible intervention would involve assigning a health system navigator to patients to provide personalized support and help coordinate their care, including ongoing clinic visits. One recently presented study showed the positive impact of frequent monitoring by dedicated nurses on mortality reduction in the early months of ART among high-risk patients initiating treatment in resource-limited settings [63].

We found that settings with higher rates of LTFU have the most to gain from LTFU prevention interventions. However, this analysis shows that even in settings with moderate LTFU rates, interventions to decrease LTFU may be cost-effective. However, it is critical to ensure that, from a programmatic perspective, decision makers not reward poor rates of follow-up with greater resources because of the perverse incentive such a policy might create. Cost-effectiveness can inform programmatic resource allocation, but should not be the only factor governing such decisions.

In sensitivity analyses, the cost of ART, particularly expensive second-line ART, affected cost-effectiveness the most. One of the determinants of the importance of the cost of second-line ART is the fact that in the base case analysis, patients continue receiving second-line ART, even after clinical failure. In this analysis we assumed that ART is continued after second-line failure for its independent protective effect, and this strategy was cost-effective. Decisions about potentially stopping ART after clinical failure, particularly in areas where drug supply may be insufficient, should be the focus of additional investigation.

As of 2007, only 5% of all people receiving ART in low- and middle-income countries were estimated to be receiving a second-line regimen [64]. However, these numbers are expected to increase greatly in future years as programs continue to be rolled out and patients have been on first-line regimens longer. The WHO recommends that countries establish national treatment guidelines with specific second-line regimens, and they specified the highest priority second-line regimen to consist of a ritonavir-boosted PI (lopinavir or atazanavir) and two nucleoside reverse transcriptase inhibitors (NRTIs, abacavir/didanosine or tenofovir/emtricitabine). In order to ensure continued access to life-saving HIV treatment, decreasing the costs of second-line ART should be a major priority.

While this analysis showed that LTFU prevention strategies carry substantial survival benefits and can be cost-effective according to international standards, it is critical to combine these interventions with programs that maximize timely linkage to care and ART initiation in patients newly diagnosed with HIV.

The CEPAC model estimate of life expectancy in HIV-infected individuals of 16.8 y (undiscounted) receiving ART may seem high at first glance, but it lies within the plausible values for Côte d'Ivoire. According to the most recent WHO life tables (2006) [65], the average additional life expectancy for someone 35–39 y old (the range that includes the mean age at ART initiation used in the current analysis) is 30.2 y, which includes both HIV-infected and HIV-uninfected individuals. This positions the CEPAC-based estimate for HIV-infected individuals well within the plausible range. In contrast, HIV-infected patients who initiate ART at an average age of 37 y with a CD4 cell count of 140 cells/µl who are lost to follow-up during the first year after ART initiation and did not return to care, have an estimated life expectancy of only 2.6 y (undiscounted).

There are several limitations to this study. First, the data are from sites in Abidjan, Côte d'Ivoire, and may not be generalizable to all West African countries or all resource-limited settings. Second, our effort to estimate the impact of adding infrastructure to treatment programs with higher LTFU rates, to achieve the lower rates seen at HIV-specific centers, may not fully capture the skills that exist at specialty centers, which allow them to achieve better outcomes. In this analysis, we did not account for any benefits associated with improved health care workers' skills beyond patient retention. More broadly, because of the lack of data on interventions to prevent LTFU and the efficacy of such interventions, this remained a “what if” analysis, with wide-ranging intervention-projected efficacies of 10%–75%. More data on the actual efficacies of LTFU prevention interventions are needed to help decision makers better understand the impact of such interventions in their treatment programs.

While in reality, characteristics of patients who are lost to follow-up may differ from those who remain in treatment, the current analysis assumed that all patients had an equal probability of being lost to follow-up, independent of age, gender, and stage of HIV disease. We also did not account for LTFU occurring more than 1 y after ART initiation, since it has been shown that the highest rates of LTFU occur soon after ART initiation.

LTFU is a critically important problem in many HIV treatment programs in resource-limited settings. We found that interventions to prevent LTFU, using specific strategies and cost data from Côte d'Ivoire, are likely to provide excellent value if they are moderately effective, and would lead to important survival benefits. The development, testing, and dissemination of effective programs to prevent LTFU from HIV treatment programs in resource-limited settings should be a major priority.

## Supporting Information

Text S1 Technical appendix.
(0.13 MB DOC)

## Abbreviations

ART: antiretroviral therapy
CEPAC: Cost-Effectiveness of Preventing AIDS Complications
GDP: gross domestic product
LTFU: loss to follow-up
OI: opportunistic infection
SD: standard deviation
YLS: year of life saved
