# Supplementary material for: Cost-Effectiveness of Preventing Loss to Follow-up in HIV Treatment Programs: A Côte d'Ivoire Appraisal
Source: PLoS Med. 2009 Oct 27;6(10):e1000173. doi: 10.1371/journal.pmed.1000173 (PMC2762030; doi:10.1371/journal.pmed.1000173)
Supplement: Text S1 — Technical appendix. (0.13 MB DOC) [file pmed.1000173.s001.doc]

**TECHNICAL APPENDIX**

*I. Opportunistic infection prophylaxis*

Patients with CD4 counts <500/µl initiated co-trimoxazole prophylaxis and continued to receive this prophylaxis as long as they remained in care [1]. Treatment with co-trimoxazole prophylaxis reduced the risk of several opportunistic infections, including bacterial diseases and malaria, but carried a small risk of drug-related toxicity [2].

*III. Deriving costs of the LTFU prevention interventions*

We used the CEPAC International model to estimate the number of primary and secondary cases of OIs occurring in the first year in a simulated population of 100,000 with characteristics similar to those of patients in the CePReF clinic. Using the pharmacy component of OI costs from the Cotrimo cost data, [2] we estimated the overall cost of OI drug costs for that year and then divided by 100,000 to get the cost per person per year and divided that number by 12 to get the monthly per person cost of OI drugs ($1.59 US, 2006).

To estimate the cost for improving health worker skills, we used the difference in CePReF cost data between the most and least expensive estimates of personnel costs ($7,570 vs. $4,005). We divided this number by the number of patients in the CePReF clinic (n=3,507) to yield a cost of $1 US/person/month. Finally, the costs of providing meals and reimbursing patients for transportation to their monthly clinic visit were each estimated at $1 US/person/month.

**Technical Appendix Table 1. Additional model input parameters for an analysis of LTFU from HIV programs in Abidjan, Côte d’Ivoire**.

| **Variable** | | **Value** | **Reference** |
| --- | --- | --- | --- |
| **Baseline Cohort Characteristics** | |  |  |
| HIV RNA distribution (%) | |  | [3] |
| >100,000 copies/ml | | 69.2 |  |
| 30,001-100,000 copies/ml | | 22.3 |  |
| 10,001-30,000 copies/ml | | 7.06 |  |
| 3,001-10,000 copies/ml | | 1.35 |  |
| 500-3,000 copies/ml | | 0.11 |  |
| <500 copies/ml | | 0.00 |  |
| **Natural history of disease** | |  |  |
| Monthly CD4 decline (cells/μl) stratified by HIV RNA (SD) | | | [4] |
| >30,000 copies/ml | | 6.4 (0.3) |  |
| 10,001-30,000 copies/ml | | 5.4 (0.2) |  |
| 3,001-10,000 copies/ml | | 4.6 (0.2) |  |
| 500-3,000 copies/ml | | 3.7 (0.2) |  |
| <500 copies/ml | | 3.0 (0.3) |  |
| Monthly risk of severe OIs (%)* | |  | [5,6] |
| Bacterial | | 0.41-3.34 |  |
| Bacterial enteritis | | 0.18-1.33 |  |
| Tuberculosis | | 0.02-0.67 |  |
| Malaria | | 1.24-2.98 |  |
| **Variable** | | **Value** | **Reference** |
| **Natural history of disease** | |  |  |
| Monthly risk of severe OIs (%)* | |  | [5,6] |
| Visceral Stage 3-4 | | 0.04-2.92 |  |
| Non-visceral Stage 3-4 | | 0.02-2.75 |  |
| Non-specific Stage 3-4 | | 0.05-2.08 |  |
| Other Non-specific | | 0.75-3.77 |  |
| Monthly risk of mild OIs (%) | |  | [5,6] |
| Bacterial | | 1.00-2.01 |  |
| Fungal^‡^ | | 0.67-9.46 |  |
| Other | | 0.88-4.14 |  |
| Monthly risk of HIV-related death (%) | | 0.00-5.19 | [5,7] |
| **CD4-dependent probability of death from OI (%)** | | | [5,7] |
| Bacterial, severe | | 0.00-14.29 |  |
| Tuberculosis | | 0.00-50.00 |  |
| Malaria | | 0.00-14.29 |  |
| Visceral Stage 3-4 | | 0.00-45.00 |  |
| Non-specific Stage 3-4 | | 0.00-45.00 |  |
| Other Non-specific | | 0.00-14.29 |  |
| **Efficacy of co-trimoxazole (% reduction in probability of occurrence of OI)** | | | [2,8] |
| Bacterial, mild | 48.8 | |  |
| Bacterial, severe | 49.8 | |  |
| **Variable** | **Value** | | **Reference** |
| **Efficacy of co-trimoxazole (% reduction in probability of occurrence of OI)** | | | [2,8] |
| Fungal, mild | -46.4‡ | |  |
| Malaria | 88.4 | |  |
| Stage 3-4 Visceral | 17.9 | |  |
| Non-specific Severe | 17.9 | |  |

OI: opportunistic infection, SD: standard deviation

*Risk of opportunistic infection varies by CD4 stratum, divided into <50/μl, 51-200/μl, 201-350/μl, 351-500/μl, >500/μl

‡Percent monthly risk of mild fungal infections is increased by 46.4% in the presence of co-trimoxazole [2,8].

**REFERENCES**

1. Toure S, Kouadio B, Seyler C, Traore M, Dakoury-Dogbo N, et al. (2008) Rapid scaling-up of antiretroviral therapy in 10,000 adults in Cote d'Ivoire: 2-year outcomes and determinants. AIDS 22: 873-882.

2. Yazdanpanah Y, Losina E, Anglaret X, Goldie SJ, Walensky RP, et al. (2005) Clinical impact and cost-effectiveness of co-trimoxazole prophylaxis in patients with HIV/AIDS in Cote d'Ivoire: a trial-based analysis. AIDS 19: 1299-1308.

3. Seyler C, Anglaret X, Dakoury-Dogbo N, Messou E, Toure S, et al. (2003) Medium-term survival, morbidity and immunovirological evolution in HIV-infected adults receiving antiretroviral therapy, Abidjan, Cote d'Ivoire. Antivir Ther 8: 385-393.

4. Mellors JW, Munoz A, Giorgi JV, Margolick JB, Tassoni CJ, et al. (1997) Plasma viral load and CD4+ lymphocytes as prognostic markers of HIV-1 infection. Ann Intern Med 126: 946-954.

5. Minga A, Danel C, Abo Y, Dohoun L, Bonard D, et al. (2007) Progression to WHO criteria for antiretroviral therapy in a 7-year cohort of adult HIV-1 seroconverters in Abidjan, Cote d'Ivoire. Bull World Health Organ 85: 116-123.

6. Anglaret X, Messou E, Ouassa T, Toure S, Dakoury-Dogbo N, et al. (2003) Pattern of bacterial diseases in a cohort of HIV-1 infected adults receiving cotrimoxazole prophylaxis in Abidjan, Cote d'Ivoire. AIDS 17: 575-584.

7. Seyler C, Messou E, Gabillard D, Inwoley A, Alioum A, et al. (2007) Morbidity before and after HAART initiation in Sub-Saharan African HIV-infected adults: a recurrent event analysis. AIDS Res Hum Retroviruses 23: 1338-1347.

8. Anglaret X, Chene G, Attia A, Toure S, Lafont S, et al. (1999) Early chemoprophylaxis with trimethoprim-sulphamethoxazole for HIV-1-infected adults in Abidjan, Cote d'Ivoire: a randomised trial. Cotrimo-CI Study Group. Lancet 353: 1463-1468.
